# Childhood Trauma Associated with Enhanced High Frequency Band Powers and Induced Subjective Inattention of Adults

**DOI:** 10.3389/fnbeh.2017.00148

**Published:** 2017-08-15

**Authors:** Seung-Hwan Lee, Yeonsoo Park, Min Jin Jin, Yeon Jeong Lee, Sang Woo Hahn

**Affiliations:** ^1^Clinical Emotion and Cognition Research Laboratory, Inje University Goyang, South Korea; ^2^Department of Psychiatry, Inje University, Ilsan-Paik Hospital Goyang, South Korea; ^3^Department of Psychology, Sogang University Seoul, South Korea; ^4^Department of Psychology, Chung-Ang University Seoul, South Korea; ^5^Department of Psychiatry, Soonchunhyang University Seoul Hospital Seoul, South Korea

**Keywords:** childhood trauma, EEG, inattention, ADHD, depression

## Abstract

Childhood trauma can lead to various psychological and cognitive symptoms. It has been demonstrated that high frequency electroencephalogram (EEG) powers could be closely correlated with inattention. In this study, we explored the relationship between high frequency EEG powers, inattention, symptoms of adult attention deficit hyperactivity disorder (ADHD), and childhood traumatic experiences. A total of 157 healthy Korean adult volunteers were included and divided into two groups using the Childhood Trauma Questionnaire (CTQ) score. The subjective inattention scores, ADHD scale, and anxiety and depression symptom were evaluated. EEG was recorded and quantitative band powers were analyzed. The results were as follows: (1) the high CTQ group showed significantly increased delta, beta1, beta2, beta3 and gamma, and significantly decreased low alpha power compared to the low CTQ group; (2) the high CTQ group had higher inattention score compared to the low CTQ group; (3) the high CTQ group had higher adult ADHD scores; (4) CTQ scores showed significant positive correlations with inattention scores, and adult ADHD scores; (5) unexpectedly, the inattention scores showed significant positive correlations with beta powers and a negative correlation with low alpha power; and (6) the moderated mediation model was confirmed: the depression fully mediated the path from state anxiety to inattention, and the CTQ significantly moderated the pathway between anxiety and depression. Our results show the possibility that childhood adversity may cause subjective inattention and adult ADHD symptoms. Depressive symptoms fully mediated the path from anxiety to inattention, especially in those who report severe childhood traumatic experiences.

## Introduction

Proper attention is essential to successfully function as a human being. However, inattention is common in daily life. Despite no objective evidence of an attentional deficiency, subjective inattention may be an important issue as people frequently complain about and consider it as an annoyance that makes life uncomfortable.

Childhood trauma has been associated with cognitive dysfunctions (Perez and Widom, 1994; Pechtel and Pizzagalli, 2011), including attention deficits in both children and adults (Hart and Rubia, 2012). Beers and De Bellis (2002) found that traumatized children with post-traumatic stress disorder (PTSD) showed an impairment in sustained attention and were more vulnerable to distractions compared to controls. Since inattention is one of the fundamental symptoms of attention deficit hyperactivity disorder (ADHD; Ford et al., 2000), the exposure to traumatic events in childhood is a risk factor for ADHD in childhood (McLeer et al., 1994; Merry and Andrews, 1994; Stevens et al., 2008) and later in adulthood (Rucklidge and Kaplan, 1997; Rucklidge et al., 2006; Szymanski et al., 2011; Prada et al., 2014; Evren et al., 2016).

The prevalence of ADHD in childhood is 3%–7% (American Psychiatric Association, 2000); symptoms of ADHD persist from childhood into adulthood in 30% to 60% of cases (Mannuzza et al., 1991; Weiss and Murray, 2003). The average prevalence rate of adult ADHD in the general population has been estimated to be 3.4% in a large cross-national study (Fayyad et al., 2007). According to previous reports (Biederman et al., 2000; Faraone et al., 2006), inattention in adults with ADHD becomes more prominent and more persistent than hyperactivity or impulsivity. Adults with ADHD continue to present attentional difficulties that tend to manifest in personal relationships and academic pursuits, and adults with ADHD often report problems with short term memory, distractibility, and impulsivity (Goldman et al., 1998; Wender, 1998). Previous research (Mann et al., 1992; Bresnahan et al., 1999) examining electrophysiological measures in adults with ADHD reported increased slow wave (delta and theta) activity compared to normal controls. The elevation in theta activity, reduced beta activity (Bresnahan et al., 1999), and higher theta/beta ratio (Lubar, 1991) were found in the frontal region in adults with ADHD. Barry et al. (2003) reported that elevated theta, reduced alpha and beta activity, and elevated theta/alpha and theta/beta ratios were the most common findings of ADHD. Roh et al. (2015) did not find any electroencephalogram (EEG) band activity difference in boys with ADHD compared to healthy participants. However, they reported that fronto-central theta activity was significantly correlated with inattention score and delayed reaction time.

Recently, we found a significant negative correlation between EEG beta and gamma power and subjective inattention in patients with major depressive disorder (Roh et al., 2016). In addition, we found that depression fully mediated the path of anxiety to inattention (Roh et al., 2016). However, anxiety did not mediate the path from depression to inattention (Roh et al., 2016). Previous studies have suggested that anxiety symptoms usually precede the development of depression. Gorman et al. (2002) suggested that generalized anxiety disorder often precedes the development of depression. Wittchen et al. (2000), in their 4–5-year prospective-longitudinal community study, concluded that most anxiety disorders are almost always primary disorders that substantially increase the risk for secondary depression.

In the present study, our aim was to examine both the relationship between past childhood traumatic experience and current adult ADHD symptoms and the relationship between high frequency EEG powers and subjective inattention symptom. In addition, we wanted to prove whether depression fully mediates the path from anxiety to inattention in drug-naïve nonclinical adult participants. We hypothesized that there would be: (1) a significant positive correlation between childhood traumatic experience and adult ADHD symptoms; and (2) significant negative correlations between high frequency EEG powers and subjective inattention. Additionally, we hypothesized; that (3) depression fully mediated the path from anxiety to inattention.

## Materials and Methods

### Participants

A total of 157 healthy Korean adult volunteers were included in this study. They were recruited from the local community through posters and flyers. The topic of the study was presented in posters and flyers, which included that this study was a clinical study about trauma and the participants were not to have histories of psychiatric treatment and any neurological disease. Participants with any history of neurological or other mental diseases were excluded from the study through initial screening interviews. Four were excluded due to insufficient epochs (less than 30) for the quantitative EEG (qEEG) analysis; hence, the final sample size was 153. The final sample consisted of 99 (64.7%) women and 54 (35.3%) men with a mean age of 27.72 years (SD = 6.39) and a range of 19–46. Participants were divided into two groups using the median score (cut off score = 40) of the childhood trauma questionnaire (CTQ): high CTQ group (*n* = 78, mean CTQ score = 51.63, range of CTQ score: 40–83) vs. low CTQ group (*n* = 75, mean CTQ score = 33.87, range of CTQ score: 28–39). The mean years of education was 14.73 years (SD = 1.66 years). Each participant signed an informed consent approved by the Institutional Review Board of Inje University Ilsan Paik Hospital prior to the study (2015-07-026-001).

### Psychological Measures

The CTQ was used to assess childhood trauma, such as emotional abuse, physical abuse, sexual abuse, emotional neglect, and physical neglect (Bernstein et al., 2003). It consists of 28 items and is assessed on a 5-point Likert scale. Its coefficient alpha in this study was 0.90. The State-Trait Anxiety Inventory (STAI) was used to evaluate both state and trait anxiety (Spielberger, 2010). It has 40 items and is assessed on a 4-point Likert scale. In this study, its coefficient alphas were 0.91 and 0.94 for state and trait anxiety, respectively. The Beck Depression Inventory (BDI) was used to measure depressive symptoms (Beck, 1972). It is composed of 21 items assessed on a 4-point Likert scale. Its coefficient alpha in this study was 0.90. The Korean version of the Adult ADHD Scale (K-AADHDS) consists of 18 items related to behavior and nine items related to inattention (Murphy and Barkley, 1996; Kim, 2003). Only the nine-item inattention scale (coefficient alpha = 0.79) was used in our study. The Korean version of Conners Adult ADHD Rating Scales (K-CAARS) was administered to measure symptoms of adult ADHD (Park et al., 2013). It consists of 42 items and is assessed on a 4-point Likert scale. In the present study, its coefficient alpha was 0.92. The order in which the psychological measures were applied was STAI, BDI, CTQ, K-AADHDS and K-CAARS.

### Electroencephalography (EEG) Acquisition and qEEG Analysis

Subjects were seated on a comfortable chair in a sound-attenuated room. Resting state EEG was recorded with subjects’ eyes open and eyes closed, for 3 min each. The EEG signal was acquired by a NeuroScan SynAmps amplifier (Compumedics USA, E1 Paso, TX, USA) with 62 Ag-AgCl electrodes (FP1, FPZ, FP2, AF3, AF4, F7, F5, F3, F1, FZ, F2, F4, F6, F8, FT7, FC5, FC3, FC1, FCZ, FC2, FC4, FC6, FT8, T7, C5, C3, C1, CZ, C2, C4, C6, T8, TP7, CP5, CP3, CP1, CPZ, CP2, CP4, CP6, TP8, P7, P5, P3, P1, PZ, P2, P4, P6, P8, PO7, PO5, PO3, POZ, PO4, PO6, PO8, CB1, O1, OZ, O2 and CB2) mounted on a Quik Cap using an extended 10–20 placement scheme. The ground electrode was located on the forehead and the reference electrode was attached to both mastoids. The vertical electrooculogram (EOG) was positioned above and below the left eye and the horizontal EOG was recorded at the outer canthus of each eye. The impedance was kept below 5 kΩ. All data were processed with a 0.1–100 Hz band pass filter at a sampling rate of 1000 Hz.

We analyzed the resting EEG data with eyes closed using CURRY 7 (Compumedics USA, Charlotte, NC, USA), which is widely used for EEG pre-processing. Gross artifacts, such as movement artifacts, were rejected by visual inspection by a trained individual with no prior information regarding the origin of the data. Artifacts related to eye movement or eye blinks were removed using the covariance analysis implemented in CURRY 7 (Semlitsch et al., 1986). After dividing the pre-processed EEG data into epochs with a length of ~2 s (2048 points), any epochs including significant physiological artifacts (amplitude exceeding ± 100 μV) at any site over all 62 electrodes were rejected. Although we did not use ICA, we are sure that artifacts have been robustly eliminated by the pre-processing analyses in this study.

For power spectral analysis, a Fast Fourier transformation (FFT) was performed on each epoch for 62 electrode channels. FFT window size was the same as the length of each epoch (2048 points) with hamming window and there was no overlap. The frequency bin size was ~0.488 Hz (1000/2048) and the number of bins was 1025. After FFT analyses for each epoch, the resultant spectral power was averaged with respect to 30 epochs. Gudmundsson et al. (2007) reported that the highest reliability was obtained with the average montage, reliability increased with epoch length up to 40 s. However, longer epochs gave only marginal improvement. Then, band power was calculated into eight frequency bands: delta (1–4 Hz), theta (4–8 Hz), alpha1 (8–10 Hz), alpha2 (10–12 Hz), beta1 (12–18 Hz), beta2 (18–22 Hz), beta3 (22–30 Hz), gamma (30–50 Hz; Aftanas et al., 2006; Zoefel et al., 2011; Milston et al., 2013; Bönstrup et al., 2016). Considering the bin size, the closest frequencies corresponding to each band were selected. Finally, the relative power of each electrode was calculated by dividing each band power by the total power of the electrode. The relative power was averaged into three regions: anterior (FP1, F3, F7, Fz, FP2, F4 and F8), middle (T7, C3, Cz, T8 and C4), and posterior (P7, P3, O1, Pz, P8, P4 and O2). The division and selection of these regions were based on previous qEEG studies (Lee et al., 2014; Markovska-Simoska and Pop-Jordanova, 2017). The relative global powers were calculated over 62 electrodes and then averaged (Gianotti et al., 2007).

### Statistical Analysis

A chi-squared test and an independent *t*-test were used to examine differences in demographic variables. A repeated-measure ANOVA, with three regions (anterior vs. middle vs. posterior) as the within-subject factor, and two groups (low CTQ vs. high CTQ) as the between-subjects factor was conducted to assess the patterns of qEEG power activity. Mauchley’s test was used to evaluate the sphericity assumption. The correction of the degrees of freedom was made using the Greenhouse–Geisser procedure (for simplicity, the uncorrected degrees of freedom have been presented). *Post hoc* Bonferroni corrected *t*-tests were conducted for between-group comparisons.

Pearson’s correlation analysis of the entire participants was performed to evaluate the relationship between the qEEG and scores on the psychological symptom scales. The bootstrap resampling technique (*n* = 5000) was used to correct multiple *t*-tests and correlations. The robustness and stability of bootstrapping were well quantified through various previous studies (Haukoos and Lewis, 2005; Ruscio, 2008; Pernet et al., 2012). In addition, bootstrap test is widely used in EEG analysis (Pernet et al., 2011; Kim et al., 2016).

Moderated mediation was assessed using Hayes and Preacher (2014) Process designed for SPSS, which includes a resampling method (bias-corrected bootstrap) with 5000 resamples to derive the 95% confidence intervals (CI) for the indirect effects (Preacher and Hayes, 2008). The moderated mediation analysis was conducted in three steps: the mediation assessment (model 4 in Process), the moderation assessment (model 1 in Process), and the moderated mediation assessment (model 7 in Process). The Sobel test to analyze the significance of the mediating effect was also applied using model 4 in Process.

The significance level was set at *p* < 0.05. Statistical analyses were performed using SPSS 21.0 (IBM Inc., New York, NY, USA).

## Results

### Demographic and Psychological Symptoms

The demographic data and psychological symptom severity are presented in Table 1. There were no significant differences in age, education level, or gender ratio between the two groups. However, there were significant differences between psychological measure scores between the two groups. The high CTQ group scored significantly higher on the CTQ, SAI, TAI, BDI, inattention and K-CAARS compared to the low CTQ group (Table 1).

**Table 1:** Comparison of participants with low and high childhood trauma questionnaire (CTQ) scores.

	Low CTQ (*N* = 75)	High CTQ (*N* = 78)	*t*	*p*
	*Mean* ± *SD* or N (%)			
Age (years)	27.28 ± 6.45	28.14 ± 6.36	−0.83	0.40
Gender				
Male/Female (ratio)	29/46 (0.63)	25/53 (0.47)		0.39*
Education (years)	14.27 ± 1.83	14.62 ± 1.72	−1.21	0.22
Height (cm)	167.11 ± 8.09	165.93 ± 7.81	0.92	0.36
Weight (kg)	62.15 ± 10.97	59.37 ± 11.92	1.45	0.13
CTQ	33.87 ± 3.42	51.63 ± 11.07	−13.52	<0.001
Emotional neglect	11.60 ± 2.88	22.00 ± 5.19	−15.41	<0.001
Physical abuse	5.88 ± 1.23	8.49 ± 3.50	−6.19	<0.001
Sexual abuse	5.25 ± 0.74	6.49 ± 2.69	−3.91	<0.001
Emotional abuse	5.40 ± 1.16	7.51 ± 3.17	−5.51	<0.001
Physical neglect	5.73 ± 1.54	7.14 ± 2.57	−4.13	<0.001
SAI	33.68 ± 7.82	39.37 ± 7.29	−4.66	<0.001
TAI	36.01 ± 9.96	43.23 ± 8.69	−4.78	<0.001
BDI	5.49 ± 3.75	10.21 ± 6.50	−5.51	<0.001
K-AADHDS–Inattention	9.32 ± 5.12	12.97 ± 5.46	−4.27	<0.001
K-CAARS	70.16 ± 14.26	80.12 ± 13.99	−4.36	<0.001

SAI, State Anxiety Inventory; TAI, Trait Anxiety Inventory; BDI, Beck Depression Inventory; K-AADHDS, Korean version of Adult ADHD Scale; K-CAARS, Korean version of Conners Adult ADHD Rating Scales. *Chi-squared test.

### Differences in qEEG Band Power between the Groups

**Figure 1:**
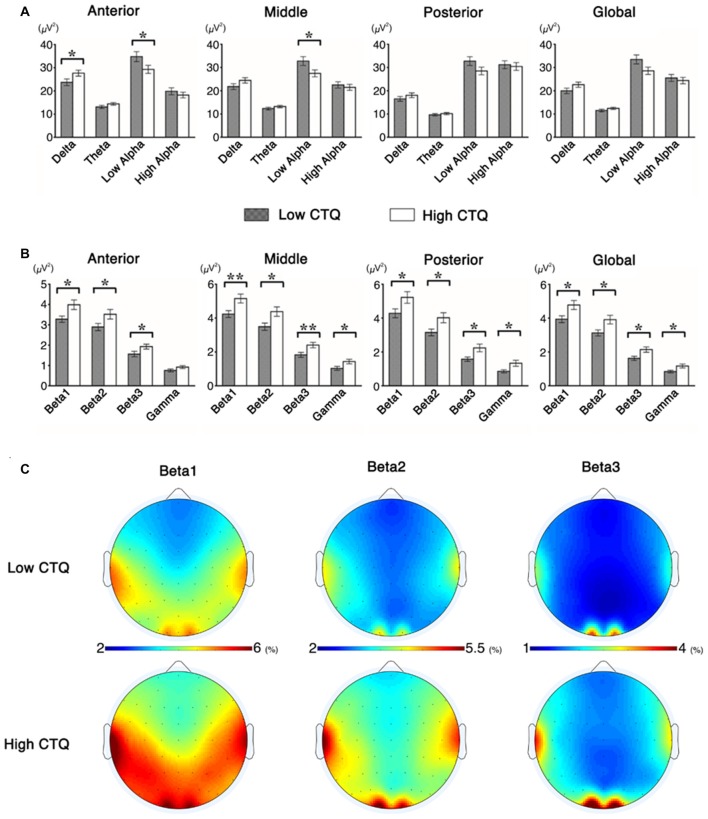
Differences in mean quantitative electroencephalogram (qEEG) indices between the groups based on Experiences of Childhood Trauma (CTQ). **(A)** Includes delta, theta, low alpha and high alpha; **(B)** includes beta 1, beta 2, beta 3 and gamma. **(C)** Scalp topographic maps of beta bands between the groups based on CTQ scores. *p < 0.05, **p < 0.01.

The delta band power showed a significant main effect for region (*F* = 255.33, df = 2, *p* < 0.001). The delta band power was the strongest at the anterior region (Figure 1A, Table 2). In addition, there was also a significant interaction between region and group (*F* = 4.91, df = 2, *p* = 0.01). The theta band power showed a significant main effect for region (*F* = 193.68, df = 2, *p* < 0.001). The theta band power was the strongest at the anterior region (Figure 1A, Table 2). The low alpha band power showed a significant main effect for region (*F* = 9.32, df = 2, *p* < 0.001), and group (*F* = 4.00, df = 1, *p* = 0.04). The low alpha band power was the strongest at the anterior region (Figure 1A, Table 2), and stronger in the low CTQ group compared to the high CTQ group. The high alpha band power showed a significant main effect for region (*F* = 281.05, df = 2, *p* < 0.001). The high alpha band power was the strongest at the posterior region (Figure 1A, Table 2). The beta 1 band power showed a significant main effect for region (*F* = 52.13, df = 2, *p* < 0.001), and group (*F* = 6.88, df = 1, *p* = 0.01). The beta 1 band power was the strongest at the posterior region, and stronger in the high CTQ group compared to the low CTQ group (Figures 1B,C, Table 2). The beta 2 band power showed a significant main effect for region (*F* = 25.04, df = 2, *p* < 0.001), and group (*F* = 6.36, df = 1, *p* = 0.01). The beta 2 band power was the strongest at the middle region, and stronger in the high CTQ group compared to the low CTQ group (Figures 1B,C, Table 2). The beta 3 band power showed a significant main effect for region (*F* = 9.78, df = 2, *p* < 0.001), and group (*F* = 7.18, df = 1, *p* < 0.01). The beta 3 band power was the strongest at the middle region, and stronger in the high CTQ group compared to the low CTQ group (Figures 1B,C, Table 2). The gamma band power showed a significant main effect for region (*F* = 15.13, df = 2, *p* < 0.001), and group (*F* = 5.84, df = 1, *p* = 0.01). The gamma band power was the strongest at the middle region, and stronger in the high CTQ group compared to the low CTQ group (Figure 1B, Table 2).

**Table 2:** Comparison of qEEG power (μV^2^) between participants with low and high childhood trauma questionnaire (CTQ) scores.

	Low CTQ (*N* = 75)	High CTQ (*N* = 78)	*t*	*p*
	*Mean* (μ*V*^2^) ± *SD*			
Anterior delta	23.73 ± 12.46	27.66 ± 11.33	−2.04	0.04
Anterior theta	13.11 ± 5.83	14.44 ± 5.02	−1.50	0.13
Anterior low_alpha	34.80 ± 18.62	29.29 ± 15.37	1.99	0.04
Anterior high_alpha	19.85 ± 12.96	18.23 ± 10.74	0.84	0.40
Anterior beta1	3.27 ± 1.34	3.98 ± 2.08	−2.49	0.01
Anterior beta2	2.89 ± 1.51	3.52 ± 2.08	−2.12	0.03
Anterior beta3	1.56 ± 1.16	1.93 ± 1.07	−2.03	0.04
Anterior gamma	0.75 ± 0.62	0.91 ± 0.66	−1.57	0.11
Middle delta	21.79 ± 10.86	24.46 ± 10.24	−1.56	0.12
Middle theta	12.33 ± 5.02	13.22 ± 4.51	−1.14	0.25
Middle low_alpha	32.76 ± 16.52	27.47 ± 13.05	2.19	0.02
Middle high_alpha	22.51 ± 11.84	21.46 ± 11.31	0.56	0.57
Middle beta1	4.23 ± 1.75	5.15 ± 2.32	−2.75	<0.01
Middle beta2	3.49 ± 1.86	4.38 ± 2.46	−2.52	0.01
Middle beta3	1.82 ± 1.25	2.40 ± 1.43	−2.64	<0.01
Middle gamma	1.03 ± 1.00	1.43 ± 1.17	−2.26	0.02
Posterior delta	16.51 ± 8.82	18.05 ± 9.06	−1.06	0.28
Posterior theta	9.64 ± 4.42	10.15 ± 4.25	−0.72	0.46
Posterior low_alpha	32.75 ± 16.28	28.52 ± 14.62	1.69	0.09
Posterior high_alpha	31.22 ± 14.54	30.45 ± 14.86	0.32	0.74
Posterior beta1	4.28 ± 2.24	5.21 ± 3.03	−2.16	0.03
Posterior beta2	3.15 ± 1.70	4.02 ± 2.61	−2.42	0.01
Posterior beta3	1.57 ± 1.10	2.23 ± 1.98	−2.54	0.01
Posterior gamma	0.85 ± 0.79	1.33 ± 1.57	−2.36	0.01
Global delta	19.96 ± 10.22	22.59 ± 9.74	−1.63	0.10
Global theta	11.51 ± 4.82	12.41 ± 4.36	−1.20	0.23
Global low_alpha	33.47 ± 16.74	28.59 ± 14.02	1.95	0.05
Global high_alpha	25.52 ± 12.98	24.39 ± 12.16	0.55	0.58
Global beta1	3.93 ± 1.73	4.77 ± 2.34	−2.50	0.01
Global beta2	3.12 ± 1.53	3.91 ± 2.25	−2.50	0.01
Global beta3	1.62 ± 1.05	2.14 ± 1.40	−2.57	0.01
Global gamma	0.83 ± 0.67	1.17 ± 1.03	−2.04	0.04

There were significant differences in qEEG band powers between the high CTQ and the low CTQ groups. In the lower band powers (Figure 1A), anterior delta was significantly higher in the high CTQ group compared to the low CTQ group (*p* = 0.04). The high CTQ group showed significantly lower anterior low alpha (*p* = 0.04) and middle low alpha (*p* = 0.02) compared to the low CTQ group. In higher band powers (Figure 1B), the high CTQ group showed significantly higher band power in anterior beta 1 (*p* = 0.01), anterior beta 2 (*p* = 0.03), anterior beta 3 (*p* = 0.04), middle beta 1 (*p* < 0.01), middle beta 2 (*p* = 0.01), middle beta 3 (*p* < 0.01), middle gamma (*p* = 0.02), posterior beta 1 (*p* = 0.03), posterior beta 2 (*p* = 0.01), posterior beta 3 (*p* = 0.01), posterior gamma (*p* = 0.01), global beta 1 (*p* = 0.01), global beta 2 (*p* = 0.01), global beta 3 (*p* = 0.01) and global gamma (*p* = 0.04) compared to the low CTQ group. Table 2 was presented for more detailed information.

### Correlation Analysis

**Figure 2:**
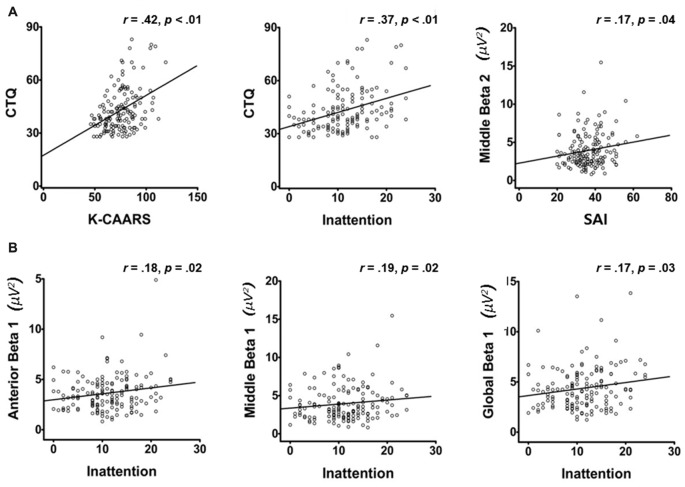
Scatterplots of psychological measurements and qEEG indices within the whole participants **(A)**. The significant correlations between inattention scores and several band powers within the whole participants **(B)**. CTQ, Childhood Trauma Questionnaire, K-CAARS, Korean version of Conners Adult ADHD Rating Scales, SAI, State Anxiety Inventory.

For the whole participants, there were significant correlations between K-CAARS scores (*r* = 0.42, *p* < 0.01), and CTQ scores and inattention scores (*r* = 0.37, *p* < 0.01; Figure 2A). The SAI score showed a significant correlation with middle beta 2 (*r* = 0.17, *p* = 0.04).

In addition, there were significant correlations between inattention scores and several band powers (Figure 2B; Table 3). The inattention scores significantly correlated with anterior low alpha (*r* = −0.16, *p* = 0.04), anterior beta 1 (*r* = 0.18, *p* = 0.02), anterior beta 2 (*r* = 0.16, *p* = 0.04), middle beta 1 (*r* = 0.19, *p* = 0.02), posterior low alpha (*r* = 0.16, *p* = 0.04), global low alpha (*r* = 0.16, *p* = 0.04) and global beta 1 (*r* = 0.17, *p* = 0.03).

**Table 3:** Correlation coefficients of qEEG indices with psychological measurements within the whole participants.

	SAI (*p*)	TAI (*p*)	BDI (*p*)	K-AADHDS-inattention (*p*)	K-CAARS (*p*)
Anterior delta	0.02 (0.78)	0.05 (0.50)	−0.06 (0.46)	0.08 (0.28)	0.03 (0.64)
Anterior theta	0.89 (0.27)	0.12 (0.11)	0.07 (0.35)	0.06 (0.45)	0.02 (0.72)
Anterior low alpha	−0.05 (0.50)	−0.12 (0.11)	−0.08 (0.31)	**−0.16 (0.04)**	−0.09 (0.25)
Anterior high alpha	−0.03 (0.64)	0.30 (0.70)	0.93 (0.25)	0.05 (0.53)	0.06 (0.41)
Anterior beta1	0.10 (0.21)	0.07 (0.34)	0.08 (0.28)	**0.18 (0.02)**	0.03 (0.71)
Anterior beta2	0.15 (0.06)	0.08 (0.29)	0.13 (0.10)	**0.16 (0.04)**	0.05 (0.48)
Anterior beta3	0.12 (0.12)	0.12 (0.13)	0.10 (0.20)	0.14 (0.07)	0.02 (0.75)
Anterior gamma	0.06 (0.45)	0.09 (0.25)	0.12 (0.12)	0.07 (0.34)	0.12 (0.88)
Middle delta	−0.02 (0.76)	0.00 (0.91)	−0.08 (0.29)	0.05 (0.50)	0.01 (0.88)
Middle theta	0.07 (0.35)	0.09 (0.26)	0.03 (0.68)	0.04 (0.60)	0.02 (0.79)
Middle low alpha	−0.03 (0.66)	−0.11 (0.15)	−0.08 (0.27)	−0.15 (0.05)	−0.10 (0.19)
Middle high alpha	−0.02 (0.71)	0.06 (0.42)	0.11 (0.16)	0.06 (0.40)	0.08 (0.29)
Middle beta1	0.06 (0.44)	0.04 (0.58)	0.07 (0.35)	**0.18 (0.02)**	0.05 (0.53)
Middle beta2	**0.16 (0.04)**	0.07 (0.39)	0.13 (0.10)	0.13 (0.10)	0.06 (0.39)
Middle beta3	0.13 (0.10)	0.10 (0.21)	0.13 (0.09)	0.07 (0.32)	0.04 (0.59)
Middle gamma	0.08 (0.32)	0.06 (0.41)	0.12 (0.13)	0.02 (0.73)	0.04 (0.57)
Posterior delta	−0.01 (0.84)	−0.00 (0.96)	−0.10 (0.19)	0.02 (0.78)	−0.01 (0.83)
Posterior theta	0.01 (0.90)	0.02 (0.77)	−0.02 (0.78)	0.00 (0.92)	0.00 (0.95)
Posterior low alpha	−0.00 (0.94)	−0.10 (0.22)	−0.05 (0.48)	**−0.16 (0.04)**	−0.10 (0.19)
Posterior high alpha	−0.00 (0.99)	0.09 (0.25)	0.12 (0.13)	0.09 (0.24)	0.09 (0.23)
Posterior beta1	−0.00 (0.96)	−0.01 (0.86)	−0.02 (0.75)	0.14 (0.07)	0.00 (0.91)
Posterior beta2	0.07 (0.34)	0.02 (0.74)	0.03 (0.70)	0.15 (0.05)	0.07 (0.37)
Posterior beta3	0.02 (0.74)	0.04 (0.61)	0.03 (0.65)	0.06 (0.42)	0.05 (0.53)
Posterior gamma	0.00 (0.94)	0.02 (0.72)	0.06 (0.45)	0.04 (0.56)	0.06 (0.43)
Global delta	−0.00 (0.91)	0.01 (0.81)	−0.08 (0.30)	0.05 (0.47)	0.01 (0.86)
Global theta	0.06 (0.45)	0.08 (0.30)	0.02 (0.72)	0.04 (0.62)	0.01 (0.82)
Global low alpha	−0.02 (0.74)	−0.11 (0.16)	−0.07 (0.37)	**−0.16 (0.04)**	−0.10 (0.21)
Global high alpha	−0.01 (0.83)	0.07 (0.37)	0.11 (0.15)	0.07 (0.34)	0.08 (0.28)
Global beta1	0.03 (0.65)	0.02 (0.80)	0.02 (0.79)	**0.17 (0.03)**	0.02 (0.78)
Global beta2	0.12 (0.13)	0.05 (0.51)	0.08 (0.30)	0.15 (0.05)	0.06 (0.42)
Global beta3	0.07 (0.38)	0.07 (0.35)	0.06 (0.42)	0.09 (0.26)	0.03 (0.66)
Global gamma	0.02 (0.74)	0.04 (0.55)	0.08 (0.32)	0.04 (0.58)	0.04 (0.59)

*SAI, State Anxiety Inventory; TAI, Trait Anxiety Inventory; BDI, Beck Depression Inventory; K-AADHDS, Korean version of Adult ADHD Scale; K-CAARS, Korean version of Conners Adult ADHD Rating Scales. Bold values mean significant correlations*.

### Moderated Mediation Analysis

The moderated mediation analysis was done in three steps: the mediation assessment, the moderation assessment and the moderated mediation assessment.

**Figure 3:**
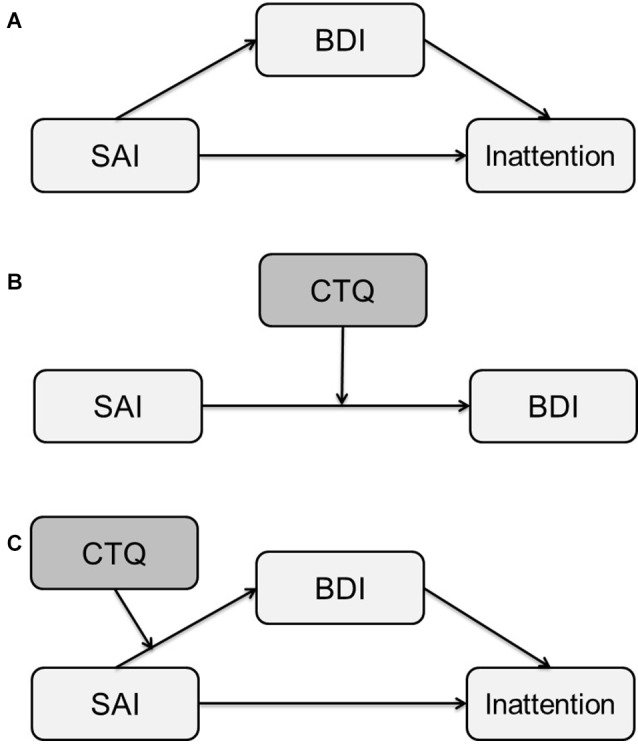
Mediation diagrams of SAI on inattention through BDI **(A)**, the moderation effect of CTQ on the pathway between SAI and BDI **(B)** and the moderated mediation model **(C)**. These analysis were conducted in the total population. SAI, State Anxiety Inventory; BDI, Beck Depression Inventory; CTQ, childhood trauma questionnaire.

First, the mediation effect of SAI on inattention through BDI in Figure 3A was analyzed. All paths were significant including the path from SAI to inattention (*B* = 0.18, *p* = 0.003), the path from SAI to BDI (*B* = 0.44, *p* < 0.001), and the path from BDI to inattention (*B* = 0.31, *p* < 0.001). The direct effect of this mediation model was significant (0.184, *p* = 0.003) and the indirect effect of this mediation model was also significant (0.135; zero does not appear in the 95% CI that lies between 0.065 and 0.209). The result of Sobel test also showed a significant mediation effect of SAI (*Z* = 3.413, *p* < 0.001). Thus, the partial mediation effect of SAI on the relationship between SAI and inattention was significant.

Second, the moderation effect of CTQ on the on the pathway between SAI and BDI in Figure 3B was examined. All coefficients of SAI (*B* = 0.34, *p* < 0.001), CTQ (*B* = 0.11, *p* = 0.002), and the interaction of SAI and CTQ (*B* = 0.01, *p* < 0.007) on BDI were significant. The moderation effect was significant because the *R*^2^ was increased due to the interaction (Δ*R*^2^ = 0.03, Δ*F* = 7.52, *p* = 0.007). People with higher CTQ score showed higher score of BDI than people with lower CTQ score. Therefore, lower score of CTQ significantly buffers the increase of depression. The moderation effect of CTQ on the on both pathways from SAI to inattention and from BDI to inattention was not significant since the coefficients of the interaction of SAI and CTQ (*B* = 0.001, *p* = 0.806) and the interaction of BDI and CTQ (*B* = −0.004, *p* = 0.296) were not significant.

Lastly, the moderated mediation model in Figure 3C was assessed. As shown in Table 4, the analysis showed the significant moderating effect of CTQ on the pathway between SAI score, the independent variable, and BDI score, the mediator. As can be seen in the Table 4, the indirect effect of BDI was significant since zero was not in the 95% CI, yet the indirect effect was higher with people with higher CTQ score.

**Table 4:** Moderated mediating effect of CTQ by BDI and significance of the moderated mediating effect.

	DV: BDI		
Variables	*B*	*SE*	*t*
(Constant)	7.51	0.37	20.15***
SAI	0.34	0.05	6.91***
CTQ	0.11	0.04	3.11**
SAI × CTQ	0.01	0.01	2.74**
	**DV: K-AADHDS-inattention**		
(Constant)	8.75	0.77	11.72***
BDI	0.31	0.08	3.69***
SAI	0.18	0.06	3.04**
CTQ	Effect	Boot *SE*	Boot Confidence Interval
*M* – 1* SD*	0.07	0.02	[0.031, 0.139]
*M*	0.10	0.03	[0.053, 0.169]
*M* + 1* SD*	0.14	0.04	[0.064, 0.224]

**p < 0.01, ***p < 0.001.

## Discussion

In this study, we assessed the relationship between high frequency EEG powers, subjective inattention symptoms, adult ADHD symptoms and childhood traumatic experience. The results were as follows: (1) the high CTQ group had higher inattention score compared to the low CTQ group; (2) the high CTQ group had higher adult ADHD score compared to the low CTQ group; (3) CTQ scores showed significant positive correlations with inattention scores, and adult ADHD scores; (4) unexpectedly, the inattention scores showed significant positive correlations with beta powers and a negative correlation with low alpha power; and (5) the moderated mediation model was confirmed: the depression fully mediated the path from state anxiety to inattention, and the CTQ significantly moderated the pathway between anxiety and depression.

### Childhood Trauma Induced Adult Inattention

We found that the high CTQ group had higher inattention score compared to that of the low CTQ group. This finding suggests that childhood trauma may affect neurocognitive problems in adulthood. Veldman et al. (2015) have investigated this topic using data from 2230 participants in a Dutch prospective cohort with a 9-year follow-up and found that childhood trauma had significant effect on attention problems in adolescence. Pçok et al. (2015) investigated this relationship in individuals with ultra-high risk for psychosis. Individuals with ultra-high risk for psychosis who had a history of childhood trauma showed worse performance in attention. They found that a history of physical trauma worsened the effect on attention.

Quidé et al. (2017) found that childhood trauma may contribute to alterations of attentional performance and the activation of the left inferior parietal lobule as a main effect of trauma exposure in participants with psychotic disorders. Additionally, a volumetric change in the brain was also found in individuals with early-life stress and childhood trauma (Spies et al., 2016). Childhood trauma was significantly correlated with attention and concentration in a negative way and the relationship was significantly mediated by decreased amygdala volume (Aas et al., 2012). In our present results, CTQ scores were significantly correlated with inattention scores. And high CTQ group showed enhanced delta, beta and gamma powers compared to low CTQ group. These findings support that childhood trauma may affect neurocognitive problems in adulthood.

### Childhood Trauma Induced Adult ADHD

We found that the high CTQ group had higher ADHD scores compared to the low CTQ group, and CTQ scores showed a significant positive correlation with adult ADHD scores. These findings suggest that childhood trauma may affect the onset of ADHD. Rucklidge et al. (2006) investigated childhood reports of abuse in adults that identified with ADHD in adulthood. For individuals with ADHD, 56% reported moderate to severe abuse or neglect in some form during childhood. This rate was much higher than that found in the controls of this study, with only 18% of female controls and 20% of male controls reporting moderate to severe abuse or neglect. In the National Epidemiologic Survey, Sugaya et al. (2012) characterized adults who reported experiences of physical abuse during their childhood. They found that child physical abuse was associated with ADHD, PTSD, bipolar disorder, panic disorder, nicotine dependance, generalized anxiety disorder, drug abuse and major depressive disorder. When ADHD was present in childhood, reports of experienced physical abuse tripled.

The strong association of child physical abuse with ADHD than with other Axis I psychiatric disorders could be related to the early onset of symptoms and highlights the impact of physical abuse during childhood. It could also be due to a bidirectional relationship in which ADHD contributes to stressful home environments that in turn provoke chronic use of violence and a later onset of other psychiatric disorders (Alizadeh et al., 2007). Lara et al. (2009) assessed childhood history of ADHD and adult ADHD in 10 countries in the World Health Organization World Mental Health Surveys. Childhood family adversities and child/adolescent exposure to traumatic events were significant risk factors in adult ADHD. Children and adults ADHD were at elevated risk for exposure to psychological trauma, but not for the development of PTSD (Ford and Connor, 2009). All this evidence supports our present results that childhood trauma may affect the onset of ADHD.

### Inattention Associated with EEG Change

Unexpectedly, we found that the inattention scores showed significant positive correlations with beta powers at anterior, middle, and global regions. For decades, it has been reported that high-frequency EEG activity is associated with attentional processes in healthy individuals and in a number of different clinical conditions. Recently, our research team Roh et al. (2016) reported that the beta (12–30 Hz) and low gamma (30–50 Hz) powers in the fronto-central regions were negatively correlated with inattention scores in patients with MDD. In a review, Barry et al. (2003) reported that elevated relative theta, reduced alpha and beta, and elevated theta/alpha and theta/beta power ratios in resting-state EEGs were the most common findings of ADHD.

However, clinical applications of EEG to ADHD patients are still a matter of debate (Loo and Makeig, 2012; Johnstone et al., 2013; Lenartowicz and Loo, 2014). Markovska-Simoska and Pop-Jordanova (2017) reported that ADHD children have increased absolute power of slow waves (theta and delta), whereas adults ADHD exhibited no differences compared with normal subjects. Ogrim et al. (2012) reported that children and adolescents with ADHD showed a positive correlation between absolute beta band power and inattention score while controls individuals showed a negative correlation between beta band power and omission errors. Hasler et al. (2016) reported that there was no relationship between cortical reactivity and conflict resolution performance in adult ADHD patients, contrasting with the association between large beta reactivity and conflict resolution slowing in control cases. They interpreted this finding as differential cortical activity patterns and alternative functional circuits in adult ADHD. The heterogeneous characteristics of target groups would be important confounding factors of this discrepancy. In the future, better designed studies controlling gender, age, anxiety level, trauma history including childhood diversity and comorbid psychiatric illness are required to solve this discrepancy.

In the present study, positive correlations were observed between inattention and beta powers. In addition, as previously mentioned, the CTQ scores were significantly correlated with anxiety score and adult ADHD scores. Our results showed that childhood trauma might be associated with high anxiety states that cause the increase of beta and gamma band powers in the brain. Those who have experienced childhood trauma portray an upward shift of the high frequency bands, which might imply unique functioning of the brain. For example, it is possible that the increased beta power of individuals with childhood trauma may reflect attentional deficits in their brain, while increased beta power of individuals without childhood trauma usually reflect healthy cognitive abilities. However, our study needs to be interpreted with caution because the correlation (*r* < 0.3) between EEG band power and psychological measures appear at a low level. In sum, our results suggest that enhanced beta power may reflect an alternative brain functioning (i.e., reduced attention), which is rarely observed in healthy controls. Further research is needed on this topic.

### Moderated Mediation Model: Depression as a Mediator and Childhood Trauma as a Moderator

We found that subjective depressive symptom (BDI) fully mediated the path from anxiety to inattention in our participants. In our previous report, the same model was confirmed in patients with MDD (Roh et al., 2016). In this previous study, anxiety explained inattention through depression, and specifically through BDI scores. We replicated our previous finding in non-clinical participants using a larger sample size. Wittchen et al. (2000) concluded that most anxiety disorders precede secondary depression in a 4–5-year prospective-longitudinal community study. Additionally, cognitive dysfunction is more severe in patients with comorbid anxiety and depressive disorder compared to patients with anxiety disorder alone (Dupuy and Ladouceur, 2008). In addition, we found a significant moderation effect of CTQ on the pathway between anxiety and depression. Our results are in line with previous findings that shows childhood trauma could develop the depressive disorder (Heim and Nemeroff, 2001). Many people could develop a state anxiety in a certain anxiety provoking situation. However, our results revealed that if they have history of childhood traumatic experiences, they could develop depressive episode by a modulating effect of it.

As a whole, the moderated mediation model was well confirmed in our participants. Our results have several meanings. First, it was the first study to prove the childhood trauma’s moderating effect among various psychological symptoms to predict inattention, which was a landmark symptom of adult ADHD. Second, the moderating effect was significant only the path from state anxiety to depression, which would be a good target for psychotherapeutic intervention. Third, our results could help to understand the whole pathological pathway about childhood trauma and adult psychopathology.

This study has some limitations. First, childhood trauma history was retrospectively reported rather than prospectively assessed and there can be some bias when recalling memory. Second, parental care style, which could be influential to an individual’s cognitive development, was not considered in this study.

In conclusion, our result showed the possibility that childhood adversity could cause subjective inattention and adult ADHD symptoms and that childhood adversity moderate the path from anxiety to depression, which path cause inattention in adulthood. Our results present insights on the relationship between childhood adversity and adult ADHD, and a different functional role of EEG beta power in adult ADHD.

## Author Contributions

S-HL suggested the idea, conducted the experiment, analyzed the results and wrote the whole manuscript. MJJ conducted the statistical analysis. YP and YJL edited the manuscript. SWH designed the study and edited the manuscript.

## Conflict of Interest Statement

The authors declare that the research was conducted in the absence of any commercial or financial relationships that could be construed as a potential conflict of interest.
